# Negative effects of urbanization on plants: A global meta‐analysis

**DOI:** 10.1002/ece3.9894

**Published:** 2023-03-31

**Authors:** Yuchen Hou, Junsheng Li, Guo Li, Wei Qi

**Affiliations:** ^1^ Institute of Ecology Chinese Research Academy of Environmental Sciences Beijing China; ^2^ State Key Laboratory of Grassland Agroecosystems, College of Ecology Lanzhou University Lanzhou China; ^3^ Command Center for Comprehensive Survey of Natural Resources China Geological Survey Bureau Beijing China

**Keywords:** abundance, meta‐analysis, plant, species richness, urbanization

## Abstract

Understanding the response of plant diversity to urbanization is essential for conserving urban biodiversity. In this paper, a meta‐analysis of 34 articles and 163 observations regarding the impact of urbanization on plant diversity was conducted. The results revealed that urbanization had marked negative effects on plants. Urbanization had positive effects on introduced species and negative effects on native species. In the subgroup analysis, we found that trees responded better to the effect of urbanization than herbs and shrubs. There was no evidence that urban size, population density, nighttime light, and GDP per capita had moderating effects on plant richness. Based on meta‐regression analyses, native species in urban areas were less affected by urbanization at lower latitudes. Overall, urbanization had a marginally negative effect on plant abundance. The effects of urbanization on plant diversity during different stages of urban development were inconsistent. Our research shows that the suburbs play a crucial role in the urbanization gradient; there, plants survive with high species richness.

## Graphical abstract

We found moderate urbanization increases plant diversity around the world. We discovered herbs were more strongly affected by urbanization than are shrubs, trees can adapt well to urbanization, and plant richness declined more significantly in the postindustrial phase. Native species in urban are less affected by urbanization at lower latitudes, and urban greenness can help plants defend the negative effects of urbanization.

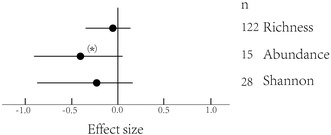

## 1 INTRODUCTION

Urbanization changes the local dominant natural ecosystem (Güneralp & Seto, 2013) and creates new ecosystems (Kowarik, 2011), ultimately increasing natural habitat fragmentation and the loss of many plants and animals (Grimm et al., 2008). With the rapid growth of urbanization worldwide (McDonald et al., 2020), the study of urban biodiversity has become increasingly important (Fischer et al., 2020; Kowarik et al., 2020; Uslu & Shakouri, 2013).

The effect of urbanization on plant richness has been widely studied, but the results have been inconsistent. Some studies indicated that urbanization reduced plant richness, with an increasing trend along urban–rural gradients (Kolbe et al., 2016; McKinney, 2002). Other studies reported a decreasing trend (Jha et al., 2019; Rija et al., 2014; Riley et al., 2018; Schwoertzig et al., 2016). A unimodal trend of plant richness along urban–rural gradients was found in some studies, which was consistent with the moderate disturbance hypothesis (Faeth et al., 2011; McKinney, 2008; Ortega‐Álvarez et al., 2011; Tian et al., 2015). Species richness varies between urban–suburban and suburban–rural areas (Alberti, 2015). There has been less research on plant abundance than on plant richness. Nock et al. (2013) showed that urban areas have a lower plant abundance, but Wang et al. (2012) found that plant abundance was negatively correlated with distance from the urban center.

To date, the only meta‐analysis of published studies focusing exclusively on plants was one that focused on the agricultural and silvicultural production of plants (Beckmann et al., 2019). In previous analyses of the effects of urbanization on biodiversity, a limited number of studies on plants were included (Beninde et al., 2015; Concepción et al., 2015; McKinney, 2008), and these were combined with studies of birds and vertebrates, which precluded the assessment of specific hypotheses related to plants.

The effects of urbanization on plants may vary among urban areas with different city sizes, climates, and land uses (Concepción et al., 2015; Faeth et al., 2011; Gerstner et al., 2014; Sari & Karasah, 2020). The characteristics of different cities, such as the geographic location of the city, history, and economics, can also impact the effect of urbanization (McKinney, 2008); these factors have been less studied (McDonald et al., 2020). Larger cities are expected to have stronger negative impacts since they accumulate higher levels of fragmentation over time (Williams et al., 2009).

In this paper, we examined studies on the impacts of urbanization on plants. Specifically, we aimed to answer the following three questions. (1) How plant richness and abundance change with urbanization? (2) Do potential species richness patterns vary among taxa? Do herbs, for example, exhibit the same pattern as shrubs or trees? (3) Do the patterns change in relation to characteristics of urbanization, such as latitude, GDP, and greenness?

## 2 MATERIALS AND METHODS

### 2.1 Study selection and meta‐analysis

We performed a systematic literature survey in the Web of Science and CNKI databases (until December 23, 2021) using the following search topics: (plant) AND (urban, suburban OR suburban, rural OR urban, rural) AND (richness OR diversity OR abundance OR density). This search identified 4313 potential studies. The literature was first filtered by title and abstract. Then, we reviewed the full text to determine whether the articles matched our criteria. Only studies published in peer‐reviewed journals were included, and we used this as the first step of quality control. The screening criteria were as follows: (1) studies that investigated plant species richness and/or abundance along urbanization gradients or spatial gradients (e.g., Batáry et al., 2018; Saari et al., 2016); (2) studies that reported means, standard deviations, and sample sizes of variation; (3) studies that included at least three spatial replicates per urbanization gradient category; and (4) studies that were published in English or Chinese.

Studies that investigated home gardens were excluded because these are directly influenced by the garden owner's preferences and do not represent the distribution of plants along the urban gradient. After the first stage of screening based on the titles and abstracts, 150 candidate studies remained. However, 116 of them were excluded after we reviewed the full text for the following reasons: fewer than three study sites in each gradient (*n* = 1); the study site was a nonpublic green space such as a home garden (*n* = 5); missing data (*n* = 27); the reported data of richness does not only include plants (*n* = 2); only some species of plants were studied (*n* = 2); the focus was on the plant's life history and traits instead of plant richness (*n* = 11); and the study was not relevant (*n* = 66).

Finally, we identified 34 studies that matched the criteria, comprising 121 observations of 32 case studies for plant richness, 28 observations of 13 case studies for Shannon–Wiener diversity, and 15 observations of eight case studies for plant abundance (The list of included studies is presented in Appendix S1).

### 2.2 Data extraction

Urban land use types were classified into four categories along the gradient: urban, suburban, rural, and natural. We used the urban land use type of the sites defined by the authors. When land use types in a study were inconsistent with these four types, we changed the terminology to fit in with our classification or amalgamated groups used in a given study into one of four categories: “natural” – natural habitats with little human interference; “rural” – very low housing density, usually farmland; “suburban” – residential areas consisting of low‐rise homes and relatively high vegetation cover; and “urban” – buildings or high‐rise residential areas (Batáry et al., 2018).

We extracted the means of richness/abundance/Shannon–Wiener index, standard deviation of variables, sample sizes, types of plants (herbs, shrubs, trees), names of cities, and population of the cities from each study. Getdata‐Graph‐Digitizer (2020) was used to obtain data when the data were presented as a graph. When the standard deviation was not reported in the study but the median and quartiles were reported, we estimated the standard deviation using the method of McGrath (2020), which is a formula‐based method based on the assumption that the outcome of the variable was normally distributed.

When the total population and population densities of the cities were not provided in the article, we used the City population database (https://citypopulation.de) to extract the population for the year in which the study was conducted. City population density, nighttime light data, GDP, urban development stage, and greenness located in the built‐up area were recorded based on the city and the year of study. The latitudes of cities were obtained from Google Earth. The nighttime light data (Li & Zhou, 2017) covered the period from 1998 to 2018 and were used to represent light pollution in the study cities. We used global artificial impervious area (GAIA) data as the urban area (Li et al., 2020). We also extracted the urban green space coverage and GDP from GHS‐BUILT database (Florczyk et al., 2019). Per capita GDP was calculated as the total GDP divided by the average annual population. The total GDP and average annual population were extracted from GHS‐BUILT database. Nighttime light data (mean) were calculated based on the artificial impervious area of the city, which was obtained from the database (Li et al., 2020). The stage of urban development was determined according to Chenery industrialization stage theory based on GDP per capita (Huang et al., 2020). Chenery classified the economic development stage of cities through GDP per capita; it is the authoritative classification standard, and the specific criteria are shown in Table 1 of “China's economic development stage and its spatiotemporal evolution: A prefectural‐level analysis” (Qi et al., 2013).

**TABLE 1:** Dataset of meta‐analyses, showing tests of moderators with corresponding residual heterogeneities *Q*, significance *p*, the degrees of freedom df, the percentage of heterogeneity among the studies *I* ^2^, and proportion of variance explained *R* ^2^.

Response	Moderators	*Q*	*p*	df	*I* ^2^	*R* ^2^
Richness (all)	**Plant types**	**620.736**	**<.001**	**60**	**97**	**.985**
	**Gradient**	**1084.259**	**<.001**	**117**	**88**	**.719**
	**Native/Introduced**	**995.150**	**<.001**	**119**	**88**	**.626**
	City size	604.939	<.001	40	85	.870
	Density	610.916	<.001	65	85	.889
	Night light index	613.874	<.001	65	85	.883
	Latitude	995.118	<.001	115	83	.461
	GDP per capita	471.942	<.001	39	88	.945
	Development group	558.679	<.001	60	87	.841
	Greenness	516.875	<.0001	35	88	.845
Richness (urban–suburban)	City size	340.756	<.001	36	81	.706
	Density	343.519	<.001	36	81	.725
	Night light index	343.580	<.001	36	79	.542
	**Latitude**	**551.730**	**<.001**	**54**	**82**	**.847**
	GDP per capita	343.000	<.001	35	81	.767
	**Development group**	**280.928**	**<.001**	**33**	**45**	**.819**
	Greenness	516.870	<.001	35	88	.845
Abundance	Gradient	6.242	0.716	9	4	.872
	Latitude	28.468	0.007	13	71	.510

*Note*: Significant moderators are indicated in bold.

### 2.3 Meta‐analysis

All statistical analyses were conducted using the metafor package (Viechtbauer, 2010) and orchard package (Nakagawa et al., 2021) in R (R Core Team, 2021). The code is presented in Appendix S2.

We used Hedges' *g* (i.e., the unbiased standardized mean difference) instead of Cohen's *d* as the measure of the effect size because it is less biased when the sample size is small, making it comparable across studies (Hedges, & Olkin, 1985). Hedges' *g* was calculated based on the standard deviation, mean, and sample sizes of plant richness, abundance, and Shannon–Wiener index of the gradient from urban to natural (e.g., urban–suburban, suburban–rural, rural–natural). Positive effect sizes indicated an increase in plant diversity along the urbanization gradient. We established hierarchically nested models because multiple effect sizes were obtained in the same study and thus could not be considered fully independent (Kambach et al., 2016). Considering that some cities were sampled multiple times (in different studies) in the meta‐analysis, we used the article and the sampled cities as nested factors.

First, we used a hierarchically random‐effect meta‐analysis model with restricted maximum likelihood (Borenstein, 2009) to investigate the overall effect sizes for total plant richness, total abundance, and Shannon–Wiener index values separately. We include the city as the first nesting factor and the study as the second nesting factor in model. This method provided an overall picture of the impact of urbanization on plant diversity. We also performed subgroup analysis on different gradients (e.g., urban–suburban, suburban–rural, rural–natural). This provided a more specific comparative analysis of the gradient. Originally, the normality test of individual coefficients and confidence intervals was based on the Z distribution; however, Knapp and Hartung (2003) improved it with a T distribution to reduce the number of unjustified significant results. Therefore, we chose the T distribution instead of the Z distribution in our model. The results of statistical analysis included the 95% confidence interval and the heterogeneity index *Q*. *Q* obeys the *χ* ^2^ distribution with df = *n*−1, where df is the degrees of freedom. The Nakagawa and Santos (2012) method was used to report *I* ^2^ and *R* ^2^, which represent the percentage of heterogeneity among the studies and proportion of variance explained. The value was considered significant if the 95% confidence interval did not include zero (Borenstein, 2009).

Second, we performed a meta‐regression analysis by considering the city size (the population of the city), city population density, nighttime light data, latitude, GDP per capita, and greenness of the city as the continuous moderators. Meta‐regression is a regression analysis of effect values at the study level, and the smallest unit (sample) of the regression is each of the studies. Since the differences in the absolute values among the moderators (e.g., city size, city population density, and GDP per capita) were large, we log‐transformed them to achieve a normal distribution. The population of cities represented the city size, and nighttime light data represented the light pollution. Most studies were carried out during the plant growing season, so we did not consider the season as a variable.

Third, we performed meta‐subgroup analysis with the stage of urban development and plant type (herb, shrub, tree) as categorical variables. We examined the interaction of subgroup and effect values with grouping factors to determine whether grouping factors were significant moderators of the heterogeneity of the findings. Among the moderators, there was between‐group heterogeneity and within‐group heterogeneity. The test for within‐group heterogeneity showed whether there was significant unexplained variance left between all effect sizes.

### 2.4 Publication bias

Studies that reported relatively high effect sizes were more likely to be published than studies that reported lower effect sizes, which could influence the results of the meta‐analysis (Borenstein, 2009). To obtain more robust results, we performed funnel plots and rank correlation tests. The more asymmetric the funnel plot is, the more likely there is substantial bias. Rank correlation tests are essentially a statistical method to test the symmetry of the funnel plot (*p* < 0.05 shows that there is publication bias).

## 3 RESULTS

**FIGURE 1:**
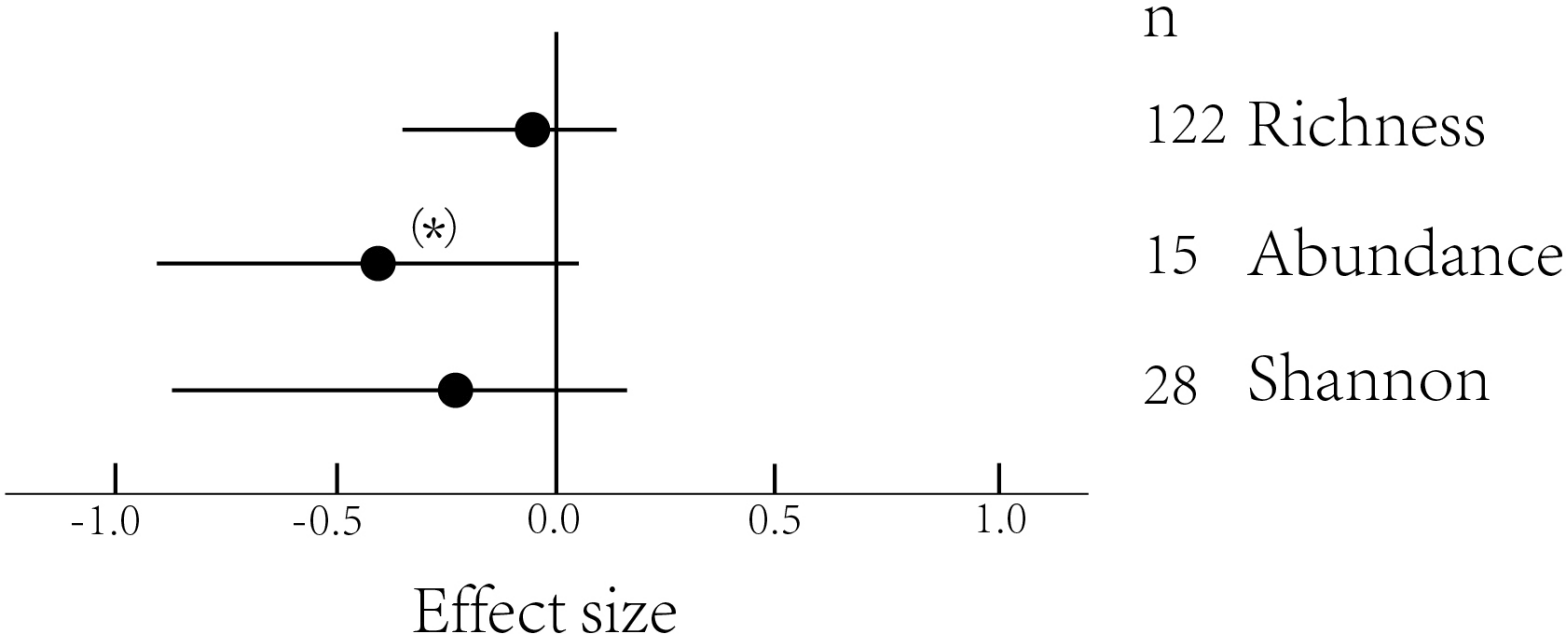
Effect sizes (Hedges' *g*) for the distribution of plant richness, abundance, and Shannon–Wiener on overall gradients. Mean effects and 95% CIs are shown. The numbers beside the labels show the sample size. Asterisks ((*) *p* < .1; *p < .05; **p < .01; ***p < .001) above the effect size symbols indicate a significant effect.

**FIGURE 2:**
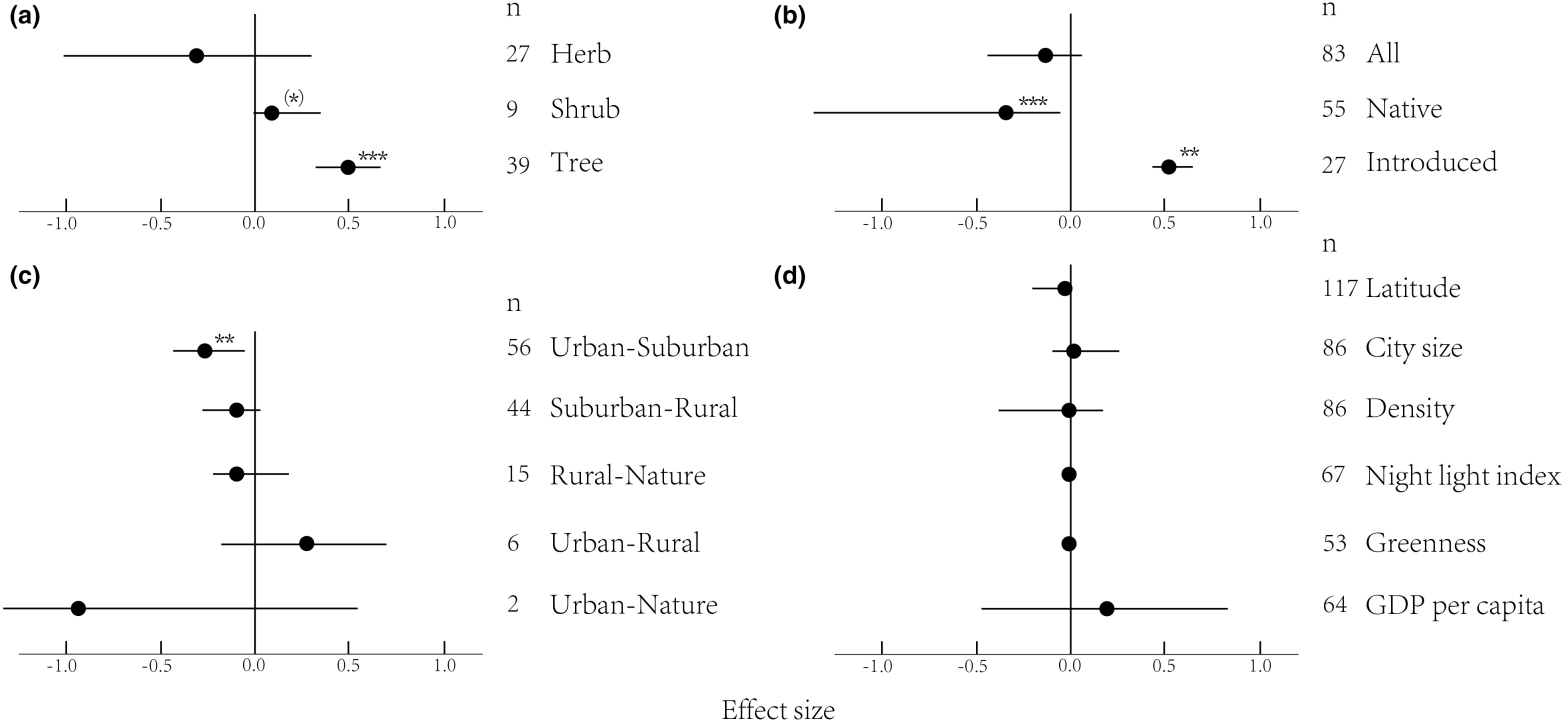
The effect of urbanization on species richness depending on life form (a), native/introduced status (b), gradients, (c) and continuous moderators (d). Mean effects and 95% CIs are shown. The numbers beside the labels show the sample size. Asterisks ((*)*p* < .1; *p < .05; **p < .01; ***p < .001) above effect size symbols indicate a significant effect.

Urbanization had an overall negative effect on plant diversity. Richness of urban–suburban areas and richness of native plants decreased with increasing urbanization (Table 1, Figures 1 and 2b). For plant abundance, there were marginally negative significant effects of urbanization (Table 1, Figure 1). The Shannon–Wiener index was not significantly influenced by urbanization (Figure 1).

There was a nonsignificant negative effect of urbanization on plant richness on the suburban–rural, rural–natural, urban–natural, and whole gradient (Table 1, Figure 2c), indicating that the negative effect of urbanization on plant richness was concentrated on the urban–suburban gradient. For the introduced/native plant richness, we observed that native plants increased along the whole gradient (Figure 2b). Contrary to the findings of native plants, introduced plants strongly decreased along the whole gradient (Figure 2b).

**FIGURE 3:**
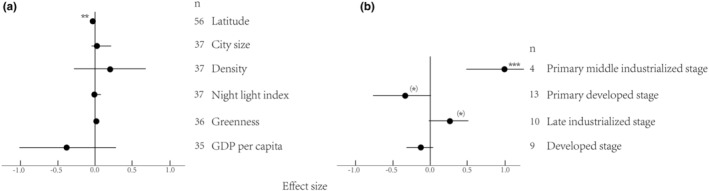
The effect of urbanization on species richness depending on continuous moderators (a) and urban development stage (b) in the urban–suburban gradient. Mean effects and 95% CIs are shown. The numbers beside the labels show the sample size. Asterisks ((*)*p* < .1; *p < .05; **p < .01; ***p < .001) above effect size symbols indicate a significant effect.

When considering tree vs. shrub and herb contrasts, urbanization had a large positive effect on trees and a less positive effect on shrubs (Figure 2a). For herbs, there was no marked moderation effect. These results, therefore, demonstrated that trees and shrubs decrease in plant richness along the urbanization gradient. Additionally, urbanization had a positive effect on plant richness in the primary middle industrialized stage and a weaker positive effect in the late industrialized stage on urban–suburban areas (Figure 3b). In the primary developed stage, urbanization had a negative effect on plant richness. These results therefore demonstrated a nonlinear decrease in species richness with the development of urban areas.

**FIGURE 4:**
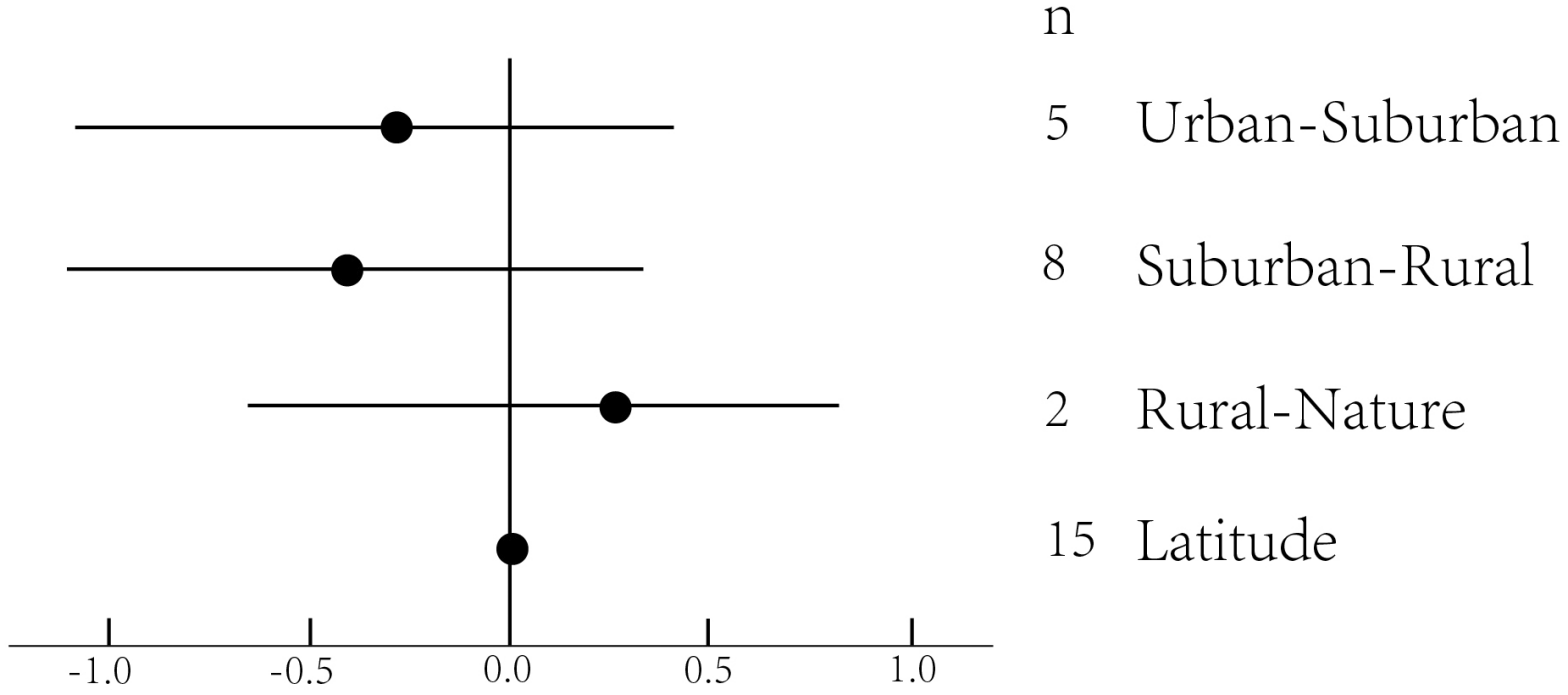
The effect of urbanization on plant abundance depending on gradients and latitude. Mean effects and 95% CIs are shown. The numbers beside the labels show the sample size. Asterisks (*p < .05; **p < .01 ***p < .001) above effect size symbols indicate a significant effect.

We found a significant moderating effect of latitude (Figure 3a) on plant richness in urban–suburban areas. There was heterogeneity in the data on plant richness, indicating the presence of unexplained variance (Table 1). There was no marked moderation effect of latitude, city size, density, night light index, greenness, or GDP per capita on the whole gradient (Figure 2d). When latitude was included as a moderator, we found a significant moderation effect of urbanization on native plant richness (Figure 3a), demonstrating that latitude affects plant richness by affecting native plants. We did not find an effect of gradients and latitude on plant abundance (Figure 4). The effects of either urbanization or the moderator on plants were on the urban–suburban gradient, not the whole gradient.

There were no significant relationships between effect sizes and sample size in the rank correlation test (*p* > 0.05). The funnel plot was basically symmetrical (Figure S1). Based on these results, we did not find evidence of publication bias in our study.

## 4 DISCUSSION

Our meta‐analysis substantiated the trend of increasing plant species richness from urban to suburban areas, confirming that urbanization had a significant effect on species richness. This result was consistent with studies by other scholars (Faeth et al., 2011; McKinney, 2008) and the response of birds and terrestrial arthropod communities to urbanization (Batáry et al., 2018; Fenoglio et al., 2020). The replacement of vegetation by urban land and the reduction in green space may be one of the main factors contributing to the reduction in biodiversity within cities (Beninde et al., 2015). The effect sizes revealed that urbanization affects plant abundance negatively, which confirms the general findings that plant abundance decreases with increasing urbanization (Broshot, 2007), although the effect values were relatively low.

The effect of urbanization varied among plant types. Trees seem to respond better to the effect of urbanization than herbs and shrubs. This may be due to two factors. One reason is that shrubs have a lower seed release height and weaker dispersal ability than trees (Pérez‐Harguindeguy et al., 2016); another is that widely planted woody ornamentals escaped. Urbanization is similar to a filter (Lopez et al., 2018), selecting out species that are more tolerant and beneficial to humans.

Additionally, the study showed opposing general trends between introduced plant species richness and native plant richness, with introduced richness decreasing and native richness increasing with decreasing urbanization. Humans introduce new plant species for landscaping and other horticultural purposes (Reichard & White, 2001), and these species spread through natural spread, transportation (von der Lippe & Kowarik, 2008), or importation as a commodity or with a commodity (Jeschke et al., 2021), which provide enormous economic value (Riley et al., 2018) and improve the well‐being benefits (Adjei & Agyei, 2015) while also resulting in biodiversity homogenization (Blouin et al., 2019; McKinney, 2006). Moreover, our data suggest that human destruction of native species exceeds the introduction of introduced species. This might be due to the reduction in anthropogenic disturbance and habitat diversity. Our results, therefore, suggest that humans directly control plants, which is caused by the constant negative and positive effects of urbanization on native and introduced plant richness.

Plant richness along the urban–rural gradient does not remain unchanged; rather, it is a dynamically changing process (Lonsdale, 1999). Urbanization has a negative effect on native plants due to drastic changes in land use. Then, humans began to restore urban ecology by introducing species that will enormously increase due to succession and reproduction processes (McKinney, 2002). The overall plant richness mostly peaked in suburban areas, which may be related to the intermediate disturbance hypothesis (Zerbe et al., 2003).

Latitude played a role in influencing the relationship between plant richness and urbanization by regulating the effects of urbanization on native plant richness in urban–suburban areas. Faeth et al. (2011) believes that the effects of urbanization vary among cities with different latitudes. We found that overall and native plant richness was higher in urban areas at low latitudes than at mid‐latitudes. These results are partly in agreement with a previous finding in an earlier synthesis on plant communities, wherein the authors reported that exotic plant species are better able to compensate for species loss in northern temperate regions. A likely explanation for this is that native species are lost more in temperate zones, resulting in more space or ecological niches for exotic species to grow. Areas at low latitudes have better hydrothermal conditions, providing different habitats for native species to tolerate urbanization (Kowarik, 2011). Most native plant species in tropical urban environments have animal vectors (Corlett, 2007) that help native plant seed dispersal (Chan et al., 2021; Deng & Jim, 2017; Diniz et al., 2019; Noreen et al., 2016; Schuttler et al., 2021). Therefore, seed spread failure may not produce the same consequence in different latitude regions (Corlett, 2007). Species that spread seeds by adhesion to animals have higher extinction risks in urban areas because the urban structure is likely to have a negative impact on butterflies, mammals, insects, and birds (Baker et al., 2003; Fenoglio et al., 2021; Hedblom & Söderström, 2010; Pignataro et al., 2020; Williams et al., 2005). Plant richness is higher at lower latitudes, where biodiversity still needs to be considered.

Plant species richness declined mainly in the primary developed stage. With growing urbanization, the consequences of changing land‐use patterns and the conversion of green spaces to other land uses (e.g., land for construction) were apparent. Since urbanization promotes introduced plant richness and reduces native plant richness, we found that studies including samples from different stages of urban development are an important reason for the different results of the study (McKinney, 2002; Rija et al., 2014; Tian et al., 2015).

The environmental factors affecting biodiversity may differ on different scales (Chase & Knight, 2013). Previous researchers found population density, nighttime lighting, and luxury affected plants on the community and county levels (Giavi et al., 2021; Leong et al., 2018; Schwartz et al., 2006). However, we did not find evidence of effects on the urban scale.

Our findings demonstrate how urbanization affects plants. Nevertheless, many ecological questions cannot be answered definitively, such as the following: When will introduced species compensate for the loss of native species after urbanization? This would require more data from cities at different stages of development or a long‐term monitoring effort for a city. The results are particularly important for urban areas and countries that are developing. We use, as in most other studies, the commonly used land use classifications (urban, suburban, rural, natural) in this article, which may raise some problems. The sampling points in some articles were not linearly distributed, which may have caused us to ignore some factors. However, we still found evidence of the effect of urbanization on plants. In future studies, it would be more helpful to use a more precise method of land use classification.

In our most comprehensive quantitative review of plants, we found that urbanization had a significant negative effect on plants. In the future, this result should be considered. We provided evidence of the importance of latitude and urban development in plant responses (which have been less found or unreported in the literature) by examining different urban areas around the world. Finally, we went one step further to explain what lies behind the differences in plant responses and found that urbanization increased the richness of introduced species and decreased the richness of native species.

## AUTHOR CONTRIBUTIONS

**Yuchen Hou:** Conceptualization (equal); data curation (equal); formal analysis (equal); investigation (equal); writing – original draft (equal). **Junsheng Li:** Conceptualization (equal); resources (equal). **Guo Li:** Conceptualization (equal); supervision (equal). **Wei Qi:** Resources (equal).

## FUNDING INFORMATION

Supported by Budget Surplus of Central Financial Science and Technology Plan (2021‐JY‐26).

## Supporting information


Appendix S1.

Appendix S2.

## Data Availability

Data are included in the Supporting Information.
