# Supplementary material for: Negative effects of urbanization on plants: A global meta‐analysis
Source: Ecol Evol. 2023 Mar 31;13(4):e9894. doi: 10.1002/ece3.9894 (PMC10065982; doi:10.1002/ece3.9894)

**Supplementary material**

**Appendix S1 List of articles included in the study**

Albrecht, Harald, and Sylvia Haider. “Species Diversity and Life History Traits in Calcareous Grasslands Vary along an Urbanization Gradient.” *Biodiversity and Conservation* 22, no. 10 (September 2013): 2243–67. <https://doi.org/10.1007/s10531-013-0437-0>.

Aronson, Myla F. J., Steven N. Handel, Inga P. La Puma, and Steven E. Clemants. “Urbanization Promotes Non-Native Woody Species and Diverse Plant Assemblages in the New York Metropolitan Region.” *Urban Ecosystems* 18, no. 1 (March 2015): 31–45. <https://doi.org/10.1007/s11252-014-0382-z>.

Blouin, Daniel, Stéphanie Pellerin, and Monique Poulin. “Increase in Non-Native Species Richness Leads to Biotic Homogenization in Vacant Lots of a Highly Urbanized Landscape.” *Urban Ecosystems* 22, no. 5 (October 2019): 879–92. <https://doi.org/10.1007/s11252-019-00863-9>.

Broshot, Nancy E. “The Influence of Urbanization on Forest Stand Dynamics in Northwestern Oregon.” *Urban Ecosystems* 10, no. 3 (August 8, 2007): 285–98. <https://doi.org/10.1007/s11252-007-0023-x>.

Chau, Ngai Lung, and Lee Man Chu. “Revegetation of Subtropical Soil Slopes: Groundcover Performance and the Implications of Urban Development and Slope Features on Plant Community.” Edited by Rob Marrs. *Applied Vegetation Science* 21, no. 4 (October 2018): 658–68. <https://doi.org/10.1111/avsc.12391>.

Cusack, Daniela F., and Taylor L. McCleery. “Patterns in Understory Woody Diversity and Soil Nitrogen across Native- and Non-Native-Urban Tropical Forests.” *Forest Ecology and Management* 318 (April 2014): 34–43. <https://doi.org/10.1016/j.foreco.2013.12.036>.

Duguay, Stéphanie, Felix Eigenbrod, and Lenore Fahrig. “Effects of Surrounding Urbanization on Non-Native Flora in Small Forest Patches.” *Landscape Ecology* 22, no. 4 (March 27, 2007): 589–99. <https://doi.org/10.1007/s10980-006-9050-x>.

Ehrenfeld, Joan G, and John P Schneider. “Chamaecyparis Thyoides Wetlands and Suburbanization: Effects on Hydrology, Water Quality and Plant Community Composition,” 2021, 25.

Fischer, Leonie Katharina, Verena Rodorff, Moritz von der Lippe, and Ingo Kowarik. “Drivers of Biodiversity Patterns in Parks of a Growing South American Megacity.” *Urban Ecosystems* 19, no. 3 (September 2016): 1231–49. <https://doi.org/10.1007/s11252-016-0537-1>.

Freitas, Érica Vanessa Durães, Maria das Dores Magalhães Veloso, and Walter Santos de Araújo. “Urbanization Alters the Composition, but Not the Diversity and Structure, of Neotropical Savanna Woody Plant Communities.” *Folia Geobotanica* 55, no. 2 (June 2020): 95–108. <https://doi.org/10.1007/s12224-020-09366-4>.

Gorosito, Cristian Andrés, and Víctor Rodolfo Cueto. “Do Small Cities Affect Bird Assemblages? An Evaluation from Patagonia.” *Urban Ecosystems* 23, no. 2 (April 2020): 289–300. <https://doi.org/10.1007/s11252-019-00915-0>.

Grella, Chiara, Adrian Renshaw, and Ian A. Wright. “Invasive Weeds in Urban Riparian Zones: The Influence of Catchment Imperviousness and Soil Chemistry across an Urbanization Gradient.” *Urban Ecosystems* 21, no. 3 (June 2018): 505–17. <https://doi.org/10.1007/s11252-018-0736-z>.

Hope, Diane, Corinna Gries, Weixing Zhu, William F Fagan, Charles L Redman, Nancy B Grimm, Amy L Nelson, Chris Martin, and Ann Kinzig. “Socioeconomics Drive Urban Plant Diversity,” n.d., 5.

Huang, Liujing, Hongfeng Chen, Hai Ren, Jun Wang, and Qinfeng Guo. “Effect of Urbanization on the Structure and Functional Traits of Remnant Subtropical Evergreen Broad-Leaved Forests in South China.” *Environmental Monitoring and Assessment* 185, no. 6 (June 2013): 5003–18. <https://doi.org/10.1007/s10661-012-2921-5>.

Huang, Liujing, Weixing Zhu, Hai Ren, Hongfeng Chen, and Jun Wang. “Impact of Atmospheric Nitrogen Deposition on Soil Properties and Herb-Layer Diversity in Remnant Forests along an Urban–Rural Gradient in Guangzhou, Southern China.” *Plant Ecology* 213, no. 7 (July 2012): 1187–1202. <https://doi.org/10.1007/s11258-012-0080-y>.

Jha, Rajeev Kumar, Nils Nölke, B.N. Diwakara, V.P. Tewari, and Christoph Kleinn. “Differences in Tree Species Diversity along the Rural-Urban Gradient in Bengaluru, India.” *Urban Forestry & Urban Greening* 46 (December 2019): 126464. <https://doi.org/10.1016/j.ufug.2019.126464>.

King, Christian M., and Stephen M. Hovick. “Wetland Plant Community Variation across Replicate Urban to Rural Gradients: Non-Native Species as Both Drivers and Passengers in Systems Impacted by Anthropogenic Land-Use.” *Urban Ecosystems* 23, no. 6 (December 2020): 1209–26. <https://doi.org/10.1007/s11252-020-01012-3>.

Kolbe, Sarah E., Arnold I. Miller, Guy N. Cameron, and Theresa M. Culley. “Effects of Natural and Anthropogenic Environmental Influences on Tree Community Composition and Structure in Forests along an Urban-Wildland Gradient in Southwestern Ohio.” *Urban Ecosystems* 19, no. 2 (June 2016): 915–38. <https://doi.org/10.1007/s11252-016-0531-7>.

Lewis, Gregory P., Abbie M. Weigel, Katherine M. Duskin, and Dennis C. Haney. “Wood Abundance in Urban and Rural Streams in Northwestern South Carolina.” *Hydrobiologia* 848, no. 18 (October 2021): 4263–83. <https://doi.org/10.1007/s10750-021-04638-2>.

Loewenstein, Nancy J., and Edward F. Loewenstein. “Non-Native Plants in the Understory of Riparian Forests across a Land Use Gradient in the Southeast.” *Urban Ecosystems* 8, no. 1 (March 2005): 79–91. <https://doi.org/10.1007/s11252-005-1420-7>.

Moffatt, S.F., S.M. McLachlan, and N.C. Kenkel. “Impacts of Land Use on Riparian Forest along an Urban – Rural Gradient in Southern Manitoba.” *Plant Ecology Formerly `Vegetatio’* 174, no. 1 (2004): 119–35. <https://doi.org/10.1023/B:VEGE.0000046055.27285.fd>.

Peng Y，Liu X H，Xue D Y，Shao X M，Jiang Y B． Effects of urbanization on indigenous plant diversity: a case study of Langfang City，China． Acta Ecologica Sinica，2012，32( 3) : 723-729．

<https://doi.org/10.5846/stxb201102140170>.

Rija, Alfan A., Abubakari Said, Kuruthumu A. Mwamende, Shombe N. Hassan, and Seif S. Madoffe. “Urban Sprawl and Species Movement May Decimate Natural Plant Diversity in an Afro-Tropical City.” *Biodiversity and Conservation* 23, no. 4 (April 2014): 963–78. <https://doi.org/10.1007/s10531-014-0646-1>.

Riley, Christopher B., Daniel A. Herms, and Mary M. Gardiner. “Exotic Trees Contribute to Urban Forest Diversity and Ecosystem Services in Inner-City Cleveland, OH.” *Urban Forestry & Urban Greening* 29 (January 2018): 367–76. <https://doi.org/10.1016/j.ufug.2017.01.004>.

Schwoertzig, Eugénie, Damien Ertlen, and Michèle Trémolières. “Are Plant Communities Mainly Determined by Anthropogenic Land Cover along Urban Riparian Corridors?” *Urban Ecosystems* 19, no. 4 (December 2016): 1767–86. <https://doi.org/10.1007/s11252-016-0567-8>.

Tian, Zhihui, Kun Song, and Liangjun Da. “Distribution Patterns and Traits of Weed Communities along an Urban-Rural Gradient under Rapid Urbanization in Shanghai, China: Weed Communities on Urban-Rural Gradient.” *Weed Biology and Management* 15, no. 1 (March 2015): 27–41. <https://doi.org/10.1111/wbm.12062>.

Vakhlamova, Tatyana, Hans-Peter Rusterholz, Victor Kamkin, and Bruno Baur. “Recreational Use of Urban and Suburban Forests Affects Plant Diversity in a Western Siberian City.” *Urban Forestry & Urban Greening* 17 (June 2016): 92–103. <https://doi.org/10.1016/j.ufug.2016.03.009>.

Vakhlamova, Tatyana, Hans-Peter Rusterholz, Yuliya Kanibolotskaya, and Bruno Baur. “Effects of Road Type and Urbanization on the Diversity and Abundance of Alien Species in Roadside Verges in Western Siberia.” *Plant Ecology* 217, no. 3 (March 2016): 241–52. <https://doi.org/10.1007/s11258-016-0565-1>.

Wang, Hong bing, Jun Qin, Bin Zhao, Jiakuan Chen, Li Dong, and Yong hong Hu. “Spatiotemporal Dynamics of Plant Diversity in Response to Farmers’ Evolved Settlements in Shanghai.” *Urban Forestry & Urban Greening* 22 (March 2017): 64–73. <https://doi.org/10.1016/j.ufug.2017.01.008>.

White, R. Jonathan, Margaret M. Carreiro, and Wayne C. Zipperer. “Woody Plant Communities along Urban, Suburban, and Rural Streams in Louisville, Kentucky, USA.” *Urban Ecosystems* 17, no. 4 (December 2014): 1061–94. <https://doi.org/10.1007/s11252-014-0376-x>.

Zhao, Min, Francisco J. Escobedo, Ruijing Wang, Qiaolan Zhou, Wenpeng Lin, and Jun Gao. “Woody Vegetation Composition and Structure in Peri-Urban Chongming Island, China.” *Environmental Management* 51, no. 5 (May 2013): 999–1011. <https://doi.org/10.1007/s00267-013-0025-9>.

Zhen Li, Zhuo-Wen Zhang, Yong-Jian Wang,Peng-Cheng Wang, Yong-Rong Xu & Zhi-Xiang Zhou. Influence of Anthropogenic Disturbances on Understory Plant Diversity of Urban Forests in Wuhan, Central China. Sains Malaysiana 41(12)(2012): 1495–1501

郭雪艳. 上海城市森林多尺度生态质量评价研究[D].华东师范大学,2017.

何宇娟. 社会经济因素和绿地管理方式对城乡梯度上植物多样性格局的影响[D].华东师范大学,2015.

**Appendix S2.** The code used in the meta-analysis

The overall analysis of plant richness and abundance with a three-level nested random-effect meta-analysis model and restricted maximum likelihood:

**rma.mv(yi,vi,method="REML", random=~1| city/study)**

The results are presented in Table 1 (“Richness”) and Fig 1.

The different gradient analyses of plant richness and abundance:

**rma.mv(yi,vi,method="REML",mods=”gradient” ,random=~1| city/study)**

The results are presented in Fig 2 and Fig 4.

Richness analysis with NTL (continuous variable, log-transformed) as a moderator:

**rma.mv(yi,vi,method="REML",mods=NTL,random=~1|city/study)**

Results presented in Fig. 2d

Richness analysis with Latitude (continuous variable) as a moderator:

**rma.mv(yi,vi,method="REML",mods=Latitude,random=~1|city/study)**

Results presented in Fig. 2d

Richness analysis with Greenness (continuous variable) as a moderator:

**rma.mv(yi,vi,method="REML",mods=average.greenness,random=~1|city/study)**

Results presented in Fig. 2d

Richness analysis with GDP (continuous variable, log-transformed) as a moderator:

**rma.mv(yi,vi,method="REML",mods=logGDP,random=~1|city/study)**

Results presented in Fig. 2d

Richness analysis with Density (continuous variable, log-transformed) as a moderator:

**rma.mv(yi,vi,method="REML",mods=logdensity,random=~1|city/study)**

Results presented in Fig. 2d

Richness analysis with city size (continuous variable, log-transformed) as a moderator:

**rma.mv(yi,vi,method="REML",mods=logpop,random=~1|city/study)**

Results presented in Fig. 2d

Richness subgroup analysis with plant types (categorical moderators)

**rma.mv(yi,vi,method="REML",mods=plant,random=~1|city/study)**

Results presented in Fig. 2b

Urban‒rural richness subgroup analysis with stage of urban development (categorical moderators)

**rma.mv(yi,vi,method="REML",** **,subset=gradient=="US",mods=~,random=~1|city/study)**

Results presented in Fig. 3a

Urban‒rural plant richness analysis with latitude (continuous variable) as a moderator:

**rma.mv(yi,vi,method="REML",** **,subset=gradient=="US",mods=~group,random=~1|city/study)**

Results presented in Fig. 3b

Calculate I²

**orchaRd::i2_ml(data)**

Calculate R²

**orchaRd::r2_ml(data)**

**Figure S1** Funnel plots reporting effect sizes and the standard errors of effect estimates


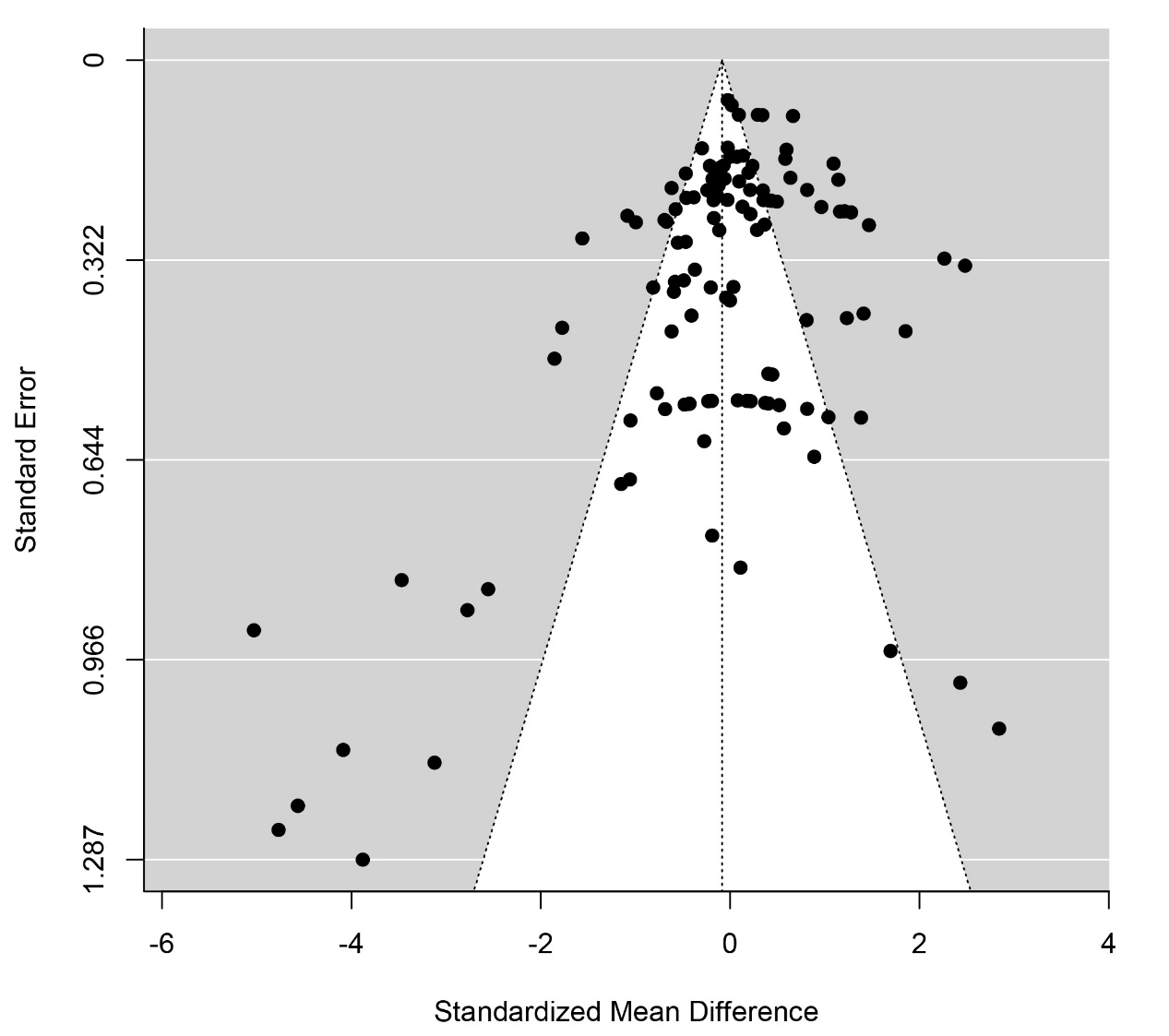

Supplement: Supplementary file 1 — Appendix S1. [file ECE3-13-e9894-s002.docx]
